# Advanced Beam Detection for Free-Space Optics Operating in the Mid-Infrared Spectra

**DOI:** 10.3390/s25196112

**Published:** 2025-10-03

**Authors:** Janusz Mikolajczyk, Waldemar Gawron, Dariusz Szabra, Artur Prokopiuk, Zbigniew Bielecki

**Affiliations:** 1 Institute of Optoelectronics, Military University of Technology, 2 Kaliskiego, 00-908 Warsaw, Poland; 2 Institute of Applied Physics, Military University of Technology, 2 Kaliskiego St., 00-908 Warsaw, Poland

**Keywords:** beam tracking, PAT, free-space optics, quadrant detector

## Abstract

The article addresses the challenges of beam position tracking in Free-Space Optical Communication (*FSOC*) systems. A review of available photodetector technologies is presented, highlighting their operating principles and applications in optical links. The analysis indicates that most current monitoring devices function with the visible and near- or short-infrared ranges. However, due to the propagation characteristics of radiation in terrestrial environments, the mid-wave infrared (*MWIR*) region offers particularly promising opportunities. To the end, the work introduces a novel detector module based on an *MWIR* quadrant detector capable of simultaneously performing two essential tasks: monitoring beam position and receiving transmitted data. Such an integrated approach has the potential to significantly simplify the design of mobile *FSOC* systems, especially those requiring accurate transceivers’ tracking. The concept was validated through laboratory experiments on an *MWIR* link model, where both the signal bandwidth and position transfer function of the quadrant detector were examined.

## 1. Introduction

The dynamic development of optoelectronics has enabled the development of sophisticated free-space optical communications (*FSOC*). *FSOC* is a line-of-sight technology that transmits information through the atmosphere using modulated optical signals. Modern commercially available systems can achieve data rates ranging from 100 Mbps to 2.5 Gbps, while experimental demonstrations employing wavelength multiplexing (WDM) have reached a bandwidth of up to 1.2 Tbps [1].

The *FSOC* systems offer numerous advantages, including high data rates, immunity to electromagnetic interference, unlicensed spectrum usage, enhanced data security, resistance to interception, protection against wiretapping, compact design, low energy consumption, easy and rapid installation, and health risks comparable or lower than those of other technologies [2]. However, their performance is strongly affected by weather conditions such as rain, fog, and turbulence. Moreover, they require high-quality stabilization of the transceiver alignment, which is why they are typically deployed in static scenarios.

To summarize, *FSOC* technology faces significant challenges in severe weather conditions and mobile communication scenarios. Various methods have been employed to mitigate weather-related effects, including optimizing the transmission spectrum, applying advanced coding techniques, and adjusting the configuration and number of transmitters and receivers [3].

The primary challenge with *FSOC* technology is ensuring availability during accidental shifts in the position of optical beams. These phenomena can result from fluctuations in the position of transceiving heads (due to wind strength or vibrations from carrier platforms) or from beam wandering caused by air turbulence. To address this, Pointing, Tracking & Acquisition (*PAT*) systems are developed, with a sensing unit that detects changes in beam positions [4].

A key component of this unit is an extra sensor that detects signal changes caused by additional reference light (beacon) or a beam splitter. Position Sensitive Detectors (*PSD*s) are often used as sensors. They provide high position resolution, fast response, a wide dynamic range, real-time position measurement, and are compact and lightweight. However, their main limitations include sensitivity to operating conditions, which can reduce accuracy, a nonlinear response, and potential reliability issues in position measurements. Additionally, some sensors have a small active area, which can significantly limit their radiation detection capabilities.

The technology proposed in this paper enables both data transmission and beam position tracking using a single detector, thereby greatly simplifying the design of *FSOC* heads and expanding their potential for mobile applications.

The analysis of state-of-the-art technology additionally indicates that *FSOC* systems typically operate in the *near-* or *short-infrared* ranges (*NIR*, *SWIR*), which broadly define the spectral range of tracking systems. However, adverse weather effects have driven growing interest in *medium- or long-wave infrared (MWIR*, *LWIR*) technologies. Several studies have highlighted the advantages of these links, particularly in terrestrial scenarios. Nevertheless, there is no available information on the use of beam position detectors operating in these spectral ranges. Therefore, the key points of this work are as follows:

- Practical verification of beam tracking capability using a quadrant detector (*QD*) in the mid-infrared spectrum (*QD-MWIR*),
- Demonstration of the potential of using a *QD-MWIR* detector for simultaneous beam tracking and data signal reception.

## 2. Study of PSD Technologies for FSOC Systems

### 2.1. Quadrant and Lateral Detectors

Optoelectronic devices are essential components and primary sources of information for beam tracking systems. Commonly used position-sensitive detectors (*PSDs*) include quadrant detectors, lateral-effect detectors (*L-PSDs*), and detector arrays [5]. Image sensors, such as CCDs, can also be employed to determine both the power level and the position of a light beam using algorithms based on photon counting or image analysis [6]. However, a major limitation of this method is the high background noise, which reduces detection performance. Furthermore, the limited frame rate and the processing time required for large pixel arrays increase the response time for beam position determination. These challenges are particularly critical for the development of simple, low-power, and compact *FSOC* devices. Therefore, the following analysis focuses exclusively on *PSD* technologies.

**Figure 1:**
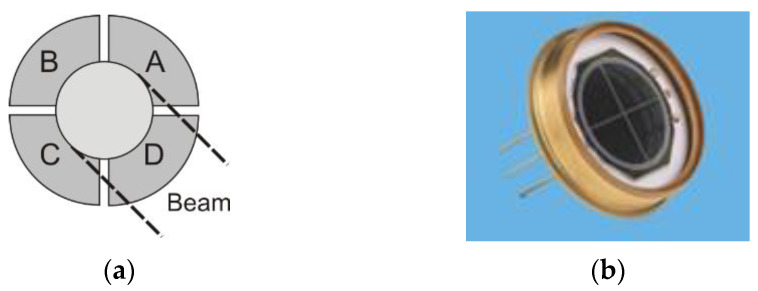
(**a**) The structure of a quadrant detector; (**b**) photo [7].

The quadrant detector measures the relative position of a light spot on its surface. Its simple design consists of four identical p-n junction photodiodes (*ABCD*) arranged symmetrically around the center, separated by narrow gaps (Figure 1). The generated photocurrents are proportional to the power of the incident beam. When the *QD* is illuminated centrally, the output currents of all four quadrants are equal. As the laser spot shifts across the detector surface, the quadrant currents vary accordingly. By analyzing the photocurrents, the position of the spot relative to the *QD* center can be determined.

Position sensing is performed by comparing the ratios of the photocurrents from the four elements. The beam position is then estimated using the conventional formula [8]:

(1) $$\hat{X}=k\frac{\left(I_{A}+I_{C}\right)-\left(I_{B}+I_{D}\right)}{I_{A}+I_{B}+I_{C}+I_{D}},\;\hat{Y}=k\frac{\left(I_{A}+I_{B}\right)-\left(I_{C}+I_{D}\right)}{I_{A}+I_{B}+I_{C}+I_{D}}$$

where $\hat{X}$ and $\hat{Y}$ represent the estimated beam position in the *x* and *y* directions, respectively. The parameter *k* is the slope constant, which depends on the beam profile. *I*_A_, *I*_B_, *I*_C_, and *I*_D_ denote the photocurrents measured in each quadrant. More advanced formulas can be applied to improve linearity in both directions and enhance the accuracy of QD-based position detection [9,10,11].

**Figure 2:**
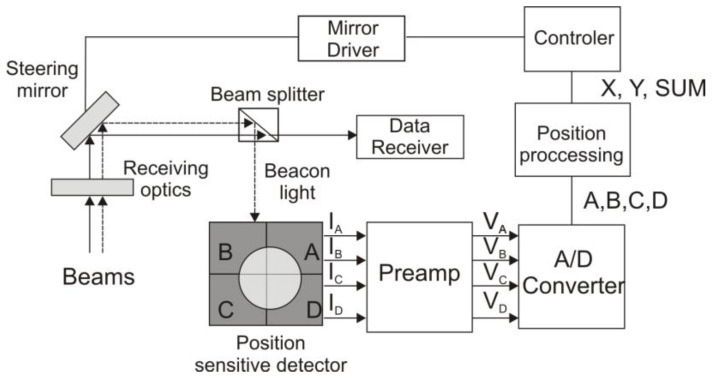
Block diagram of a typical satellite terminal showing the beacon channel with digital values of each quadrant’s signal (A, B, C, D), position coordinates (X, Y), and the summarized detector signal (SUM) (adopted from [11]).

Figure 2 shows the block diagram of the integrated beam position unit with a quadrant detector. Light passing through the interference filter is collected and focused by a lens onto the *QD,* which detects photons and generates photocurrents corresponding to the spot position. These photocurrents are then converted into voltage signals (*V*_A_, *V*_B_, *V*_C_, and *V*_D_) and processed using a dedicated configuration of sum and difference voltage amplifiers.

As a result, three voltage signals (*E*_x_, *E*_y_, and *SUM*) are generated. The *SUM* signal compensates for light fluctuations caused by instabilities in the radiation source or atmospheric effects such as scintillations. Figure 2 shows the schematic of the beam position unit [11]. The voltage signals represent the horizontal *E*_x_ and vertical *E*_y_ deviation of the transmitter position, respectively:

(2) $$E_{x}=\left(V_{B}+V_{D}\right)-\left(V_{A}+V_{C}\right);\;E_{y}=\left(V_{A}+V_{B}\right)-\left(V_{C}+V_{D}\right);\;E_{SUM}=V_{A}+V_{B}+V_{C}+V_{D},$$

and are converted into digital formats.

The *PSD* output data are used to control other parts of the *PAT* system, ensuring proper *FSOC* beam alignment. The system employs two optical beams: the first is dedicated to data transmission, while the second (a beacon beam) provides a fixed reference position for the data beam.

The position accuracy of a laser beam is limited by photon noise, background noise, and dark current noise. For the *x*-axis, this uncertainty can be expressed by [11]

(3) $$∆x=erfc\left(\frac{\sqrt{2x_{0}}}{\omega }\right),$$

where *erf* is the error function, ω is the beam radius, and x_0_ is the beam’s central position. The variance of Δ*x* equals

(4) $$\sigma_{∆x}^{2}=\frac{1+{erfc}^{2}\left(\frac{\sqrt{2x_{0}}}{\omega }\right)}{SNR}$$

The uncertainty of the beam position is therefore inversely proportional to the signal-to-noise ratio. Certain processing methods applied to *PSD* output signals can further reduce this uncertainty, for example, by employing Kalman filter-based signal filtering [12].

The lateral PSD serves the same function in *PAT* devices; however, certain differences may affect its application compared to quadrant detectors. The *L-PSD* determines beam position by measuring photocurrent flowing through resistive elements. Two design variants exist: Duolateral (*DL-PSD*) and Tetralateral (*TL-PSD*) [13]. The *DL-PSD* incorporates a resistive layer at both the anode and cathode junctions of the photodiode. This configuration separates the detector’s x- and y-position signals, providing high linearity and precise transfer characteristics. However, the additional resistive layers significantly increase manufacturing costs.

In contrast, the *TL-PSD* employs a single resistive layer with a common cathode and anode on each side of the detection area. While this design reduces production costs, it compromises response linearity, particularly away from the detector center. This decline results from the placement of the anodes along the detector edge [14]. The electrostatic field in the detector generated by the charge *Q* is equal to [15]

(5) $$\vec{E}\left(r\right)=\frac{Q}{4\pi \varepsilon r^{2}}\vec{r},$$

where ε is the dielectric constant of the medium and *r* is the distance from the charge *Q*. For boundary conditions extending to infinity, the potential at distance *r* is given by

(6) $$V\left(r\right)=\frac{Q}{4\pi \varepsilon r^{2}}.$$

**Figure 3:**
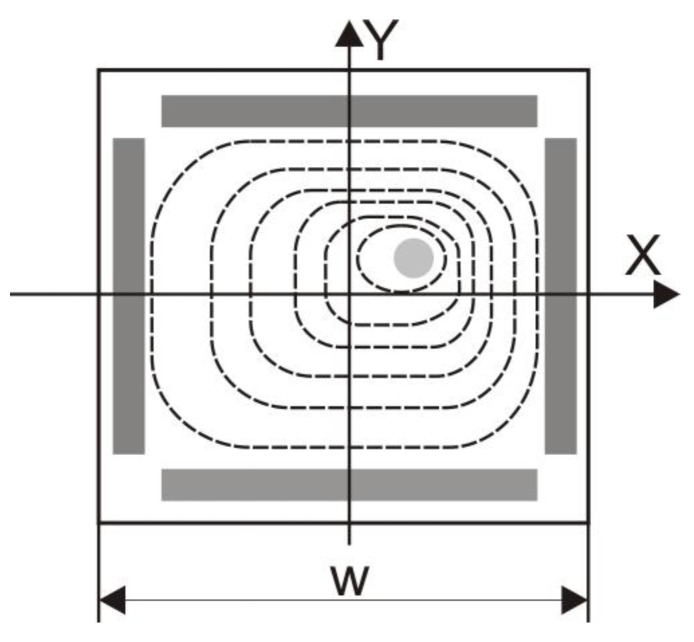
Equipotential line distribution in an *L-PSD* detector [15].

The equipotential lines have a circular shape around the charge *Q*. However, the conducting anodes that define the boundary conditions distort the field, resulting in square-shaped field lines (Figure 3).

The formula gives the potential difference in the x direction:

(7) $$∆V_{x}=\frac{Q}{4\pi \varepsilon r}\left(\frac{1}{\frac{w}{2}+x}-\frac{1}{\frac{w}{2}-x}\right)=\frac{Q}{4\pi \varepsilon r}\left(\frac{4x}{w^{2}-4x^{2}}\right).$$

where *w* is the distance between the anodes, and for i *x* << *w*, this difference is proportional to *x*. In the region near the detector center, Equation (7) can be further approximated by

(8) $$∆V_{x}=-\frac{Q}{4\pi \varepsilon w^{2}}x.$$

**Figure 4:**
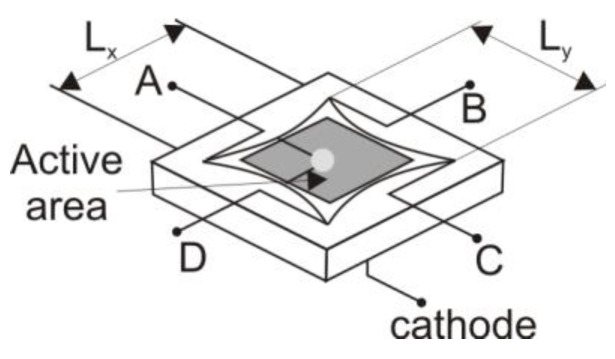
Edge shape of the *PSD* detector as presented by Thorlabs [16].

In practice, several methods are used to enhance the performance of these detectors. For example, Thorlabs Company describes a specialized edge design of detectors to improve linearity (Figure 4).

The corresponding distances in both directions are determined:

(9) $$x=\frac{L_{x}(E_{X})}{{2E}_{SUM}},$$

(10) $$y=\frac{L_{y}(E_{X})}{2E_{SUM}},$$

where *x* and *y* denote the distances from the detector center to the spot position, and *L*_x_ and *L*_y_ represent the lengths of the detector edges. It should be noted that the edge dimensions differ from the active area. Unlike *QD* detectors, *L-PSD* detectors provide information about the spot position independently of the spot shape and size, as long as the light reaches the detector. The position resolution (Δ*R*) depends on both the edge lengths and the signal-to-noise ratio, as expressed by

(11) $$∆R=L_{x}\left(\frac{V_{n}}{E_{SUM}}\right),$$

where *V*_n_ is the output noise voltage.

### 2.2. Detectors Comparison

Analysis of *PSD* detector properties shows that lateral detectors are more suitable for precise, continuous position measurements, but they are generally more expensive and complex. Quadrant detectors, on the other hand, are more straightforward, more affordable, and more robust, making them well-suited for applications where ultra-high precision and continuous tracking are not critical. Table 1 summarizes the key characteristics of these technologies.

Currently, numerous *PSD* detectors are available on the market for beam tracking applications. Table 2 presents a comparison of several models and their key performance characteristics.

In summary, the primary function of beam tracking systems is to maintain the optical alignment between the transmitting and receiving paths. This alignment can be disrupted by unintentional shifts in the position of the transceiver heads, resulting from ground vibrations, temperature fluctuations, wind effects, or atmospheric turbulence. To compensate for these disturbances, *PAT* systems are employed in practice for both coarse and fine tracking. In coarse tracking, the position signal is typically derived from inertial sensors, GPS, or cameras, whereas fine tracking most often relies on *PSD* detectors to provide high accuracy and fast response. Consequently, the following analysis focuses on this class of detectors.

### 2.3. Position-Sensitive Detectors in FSOC

In principle, identifying the key *PSD* detector technologies used in *FSOC* systems is a challenging task, with the primary criterion being their availability. Table 3 presents data on additional examples of *PSD*-based systems reported in the literature.

In practice, such a rapid analysis of position changes is not crucial given the time scales of the phenomena that can cause beam position changes and the response times of the devices used to compensate for these shifts [31]. Fluctuation rates of several Hz characterize the air turbulence that may occur, while the actuator systems operate at around several kHz. An example analysis of requirements for fine beam steering mechanisms for free-space optical system communication on satellites was presented in [32].

In the literature, several concepts of advanced *PSD* technologies dedicated to *FSOC* systems have also been described. For example, Patent EP 3 469 740 B1 presents a design with five sensitive surfaces, where a quadrant detector monitors changes and the fifth surface receives data signals. A simplified version of this approach is described in Patent No. US 9,810,862 B2, where instead of the detector element, a special “hole” in the array structure serves as the entrance aperture for the photodetection module.

**Figure 5:**
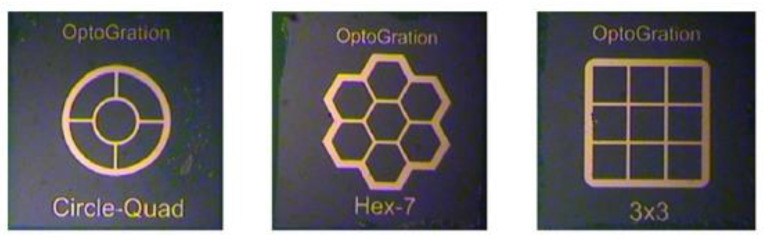
Geometries of three InAlAs/InGaAs *APD* arrays.

A similar design is described in detail in [33]. It employs InAlAs/InGaAs *APD* arrays with various geometries (Figure 5). Each array consists of elements 100 µm in diameter, with a minimum optical fill factor of 80%. In laboratory tests using a 1550 nm laser, the central pixel of the circle-quad configuration exhibited a sensitivity of −42.2 dBm for a bit error rate of 2 × 10^−9^ and a data rate of 155 Mbps.

A comparison of current *PSD* technologies can be made according to several criteria. The most straightforward is cost, where detectors operating in the *NIR* or *SWIR* range are generally more affordable. The higher price of *MWIR* detectors stems from both their more complex manufacturing process and from still relatively limited market demand. From an application perspective, however, systems operating in the *MWIR* band can provide significant advantages, including longer operational ranges and a greater number of transmission channels under conditions of reduced visibility or atmospheric turbulence. These capabilities are often critical in terrestrial communication systems as well as in data links for ground-based platforms and airborne systems. The application-related benefits of this technology have already been documented in the literature and will not be discussed here in detail [34,35,36,37,38].

## 3. Materials

*QM-4* is a multi-channel infrared (*IR*) detection module incorporating a photovoltaic, four-element quadrant geometry detector based on mercury cadmium telluride (*MCT*) heterostructure grown using the MOCVD technique (AIXTRON, Herzogenrath, Germany).

To achieve a module frequency bandwidth of up to 100 MHz, a dedicated detecting wafer architecture was developed (Table 4).

**Figure 6:**
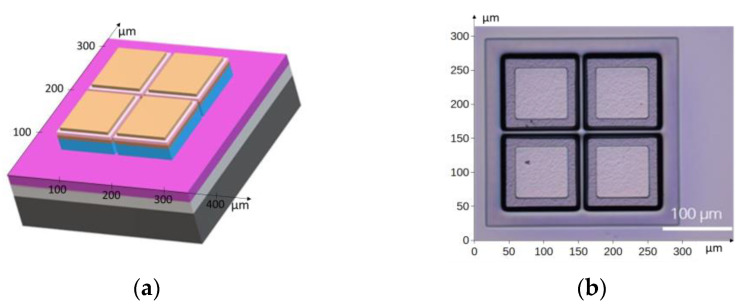
(**a**) Schematic diagram of the four-channel photodiode used in the *IR* detection module; (**b**) photograph of the edged mesa structure.

The four-segment photodiode geometry was fabricated using wet etching. The structure and the corresponding edge heterostructure image are shown in Figure 6a and Figure 6b, respectively.

A (111)-oriented Hg1−xCdxTe heterostructure, containing a lightly doped p-type narrow-gap absorber with the material composition x = 0.312, optimized for position detection of about the 4 μm radiation at 210 K and sandwiched between wide-gap, heavily doped contact layers [39], was grown by metalorganic chemical vapor deposition (MOCVD) on a CdTe-buffered semi-insulating (100) GaAs substrate [40,41]. The structure was doped with iodine and arsenic, incorporated in situ during growth, as stable and well-behaved donor and acceptor dopants, respectively. The absorber thickness was reduced approximately 0.8 μm to increase the differential impedance of this large MWIR photodiode.

Due to the internal reflection from the top of the detector mesa—covered with the indium electrode—the input radiation beam essentially passed through the absorber twice. Photodetector fabrication involved optical and electrical characterization of the epi-wafers, photolithography, mesa etching, sidewalls passivation, contact metallization, dicing into chips with four-element devices, indium bump bonding to sapphire carriers with metal leadouts, mounting on Peltier coolers with cold fingers, and sealing of the detector elements in packages filled with xenon or xenon/krypton mixtures. For practical use, the TO-8-based detector packages and associated electronics are integrated into detection modules. While the impact of the *MCT* heterostructure on noise was described in works [42,43,44,45], an analysis of its effect on crosstalk and noise lies beyond the scope of this manuscript.

## 4. Results

### 4.1. Characterization of the QD-MWIR Detector

**Figure 7:**
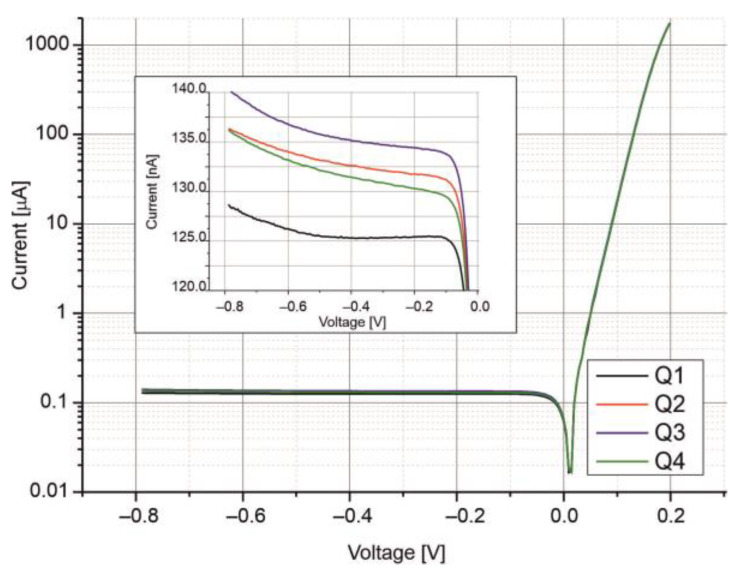
Current–voltage characteristics of the four-channel photodiode measured at T = 210 K. *Q*1, *Q*2, *Q*3, and *Q*4 represent the individual segments of the photodiode.

Figure 7 presents the dark current–voltage (*I*–*V*) characteristics of the four-segment photodiode measured at *T* = 210 K. All the segments exhibit similar *I*–*V* behavior, indicating low series resistance and nearly voltage-independent saturation currents under reverse bias. As shown in the inset of Figure 7, the saturation currents are approximately 135 nA, with a uniformity better than 10% across the segments.

**Figure 8:**
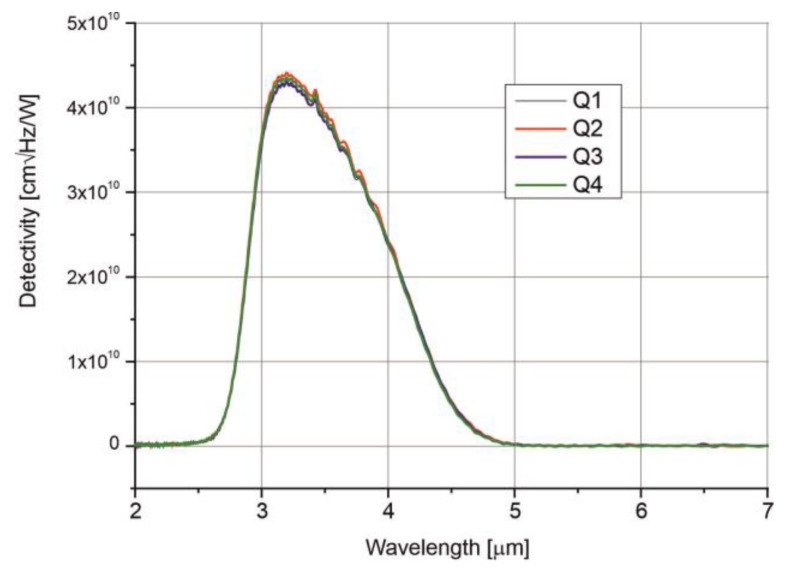
Detectivity spectra of the four-channel photodiode measured at T = 210 K.

Figure 8 presents the detectivity (D*) spectra of the four-segment photodiode, measured at a temperature of 210 K. The spectra are uniform, with a 10% cut-on at 2.7 μm and a 10% cut-off at 4.6 μm, reaching a peak detectivity at 3.2 μm.

The detectivity of each channel reaches D* = 4.3⋅× 10^10^ J at the peak wavelength and D* = 2.4⋅× 10^10^ J at the optimal wavelength.

**Figure 9:**
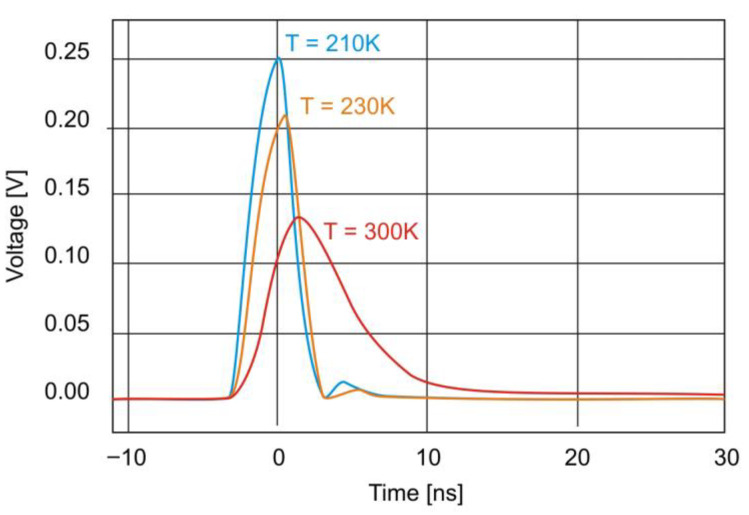
Temporal response of the photodiode at zero bias for three different temperatures.

Figure 9 shows the photodiode’s time response to 5 μm laser excitation at zero bias at three different temperatures. As the temperature decreases, the photodiode’s bandwidth increases (rise time decreases), reaching 50 MHz at T = 300 K, 200 MHz at T = 230 K, and 260 MHz at T = 210 K.

**Figure 10:**
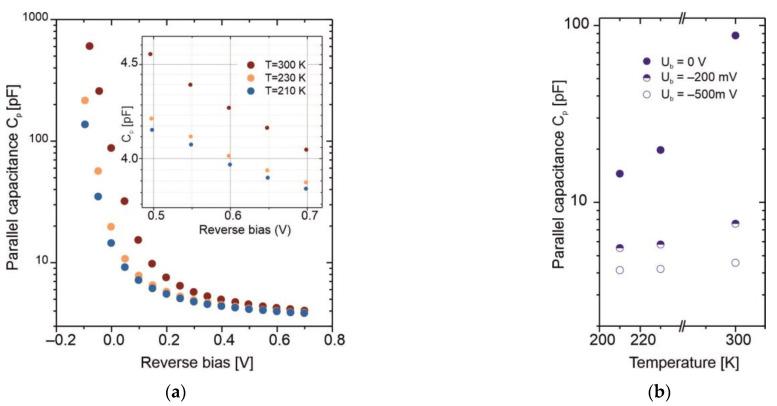
(**a**) Capacitance–voltage characteristics at various temperatures; (**b**) parallel capacitance as a function of temperature for three reverse bias voltages.

Figure 10 presents the capacitance–voltage characteristics measured at three different temperatures using an Agilent E5061B network analyzer and a specialized test fixture designed for TO-8 reflection coefficient measurement. The measurement frequency range was set from 1 kHz to 3 GHz. The capacitance is extracted by fitting the broadband reflection coefficient data to a model consisting of four parameters: series resistance *R*_S_, parallel resistance *R*_P_, parallel capacitance *C*_P_, and series inductance *L*_S_, forming a broadband equivalent circuit of the photodiode. As shown in Figure 10a, the *C*_P_ value strongly depends on the applied reverse bias. Even a low reverse voltage causes the capacitance to decrease significantly, stabilizing at a few picofarads. The temperature also has a noticeable impact—particularly at zero bias—where *C*_P_ decreases by nearly an order of magnitude, as illustrated in Figure 10b.

### 4.2. Research on the QD-MWIR Detection Module

**Figure 11:**
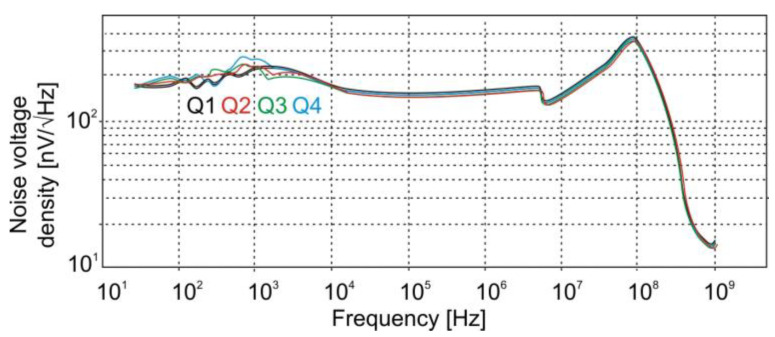
Frequency dependence of the noise voltage density for the four channels of the *QM-4* detection module.

The segmented photodiode was integrated with the DC-coupled, four-channel preamplifier and a thermoelectric cooler controller. The reverse bias voltage applied to the diode was *U*_b_ = −500 mV. The integrated detection module exhibits a voltage responsivity of *R*_V_ = 2.75 × 10^4^ V/W at 4 μm, and a detectivity of D* = 1.8 × 10^9^ J. Its low- and high-cutoff frequencies range from 1 kHz to 100 MHz. As shown in Figure 11, each channel maintains an output noise density below 160 nV/√Hz at f = 10 MHz.

### 4.3. Beam Tracking Operation

**Figure 12:**
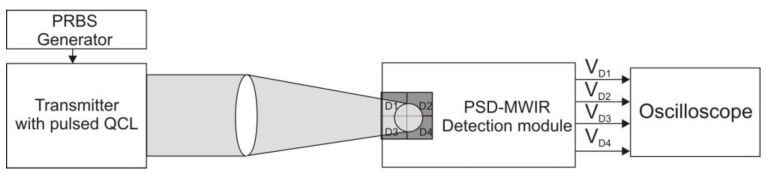
Laboratory setup for testing the *PSD-MWIR* detection module.

The functionality of the QD-MWIR detection module as a beam tracking device was verified in a laboratory setup, as shown in Figure 12. The transmitter utilizes a pulsed quantum cascade laser (model QCL834) developed at the Institute of Microelectronics and Photonics (Łukasiewicz Research Network). The laser is housed in a hermetically sealed HHL casing, which also contains a TEC module and a temperature sensor. Radiation is emitted through an optical window and has a divergence of 35^0^ (full-width at half maximum). The system generates radiation pulses at a wavelength of approximately 3.9 µm, with a peak power of up to 600 mW and a duty cycle of less than 5%. The operating point of the laser is controlled by adjusting the current and temperature using the LDP-V 03-100 V4 from Picolas and the TEC WTC32ND 2.2 A from Wavelength Electronics, respectively. The light signals are triggered by the PICOSECOND model 12,000 generator, which, during the initial phase of the research, was operated in pulse mode with adjustable duration and frequency.

**Figure 13:**
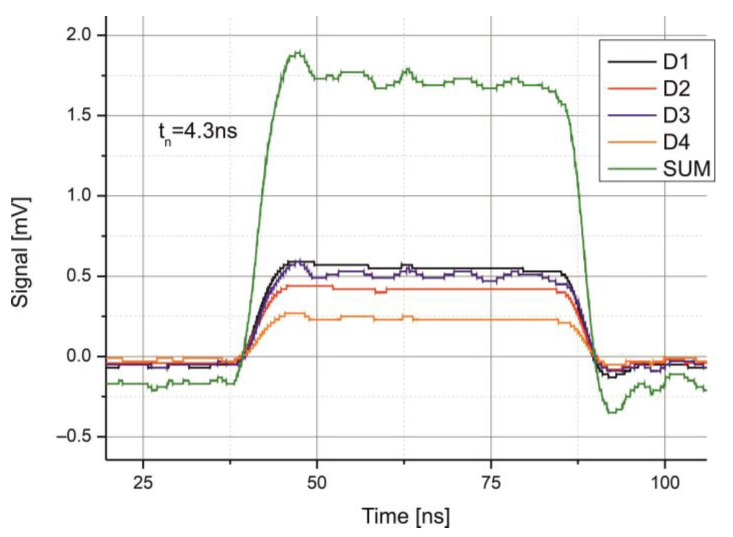
Recorded signals from each segment of the *QD-MWIR* module and their combined sum.

The pulse from each *QD*’s channel was recorded to determine the signal bandwidth. The rise time ranged from 4.1 ns to 4.4 ns. Figure 13 shows the pulse shapes of each channel as well as their combined signal. Considering the laser pulses’ rise time of 3 ns, the approximate bandwidth of the *QD-MWIR* module is estimated to be around 110 MHz.

**Figure 14:**
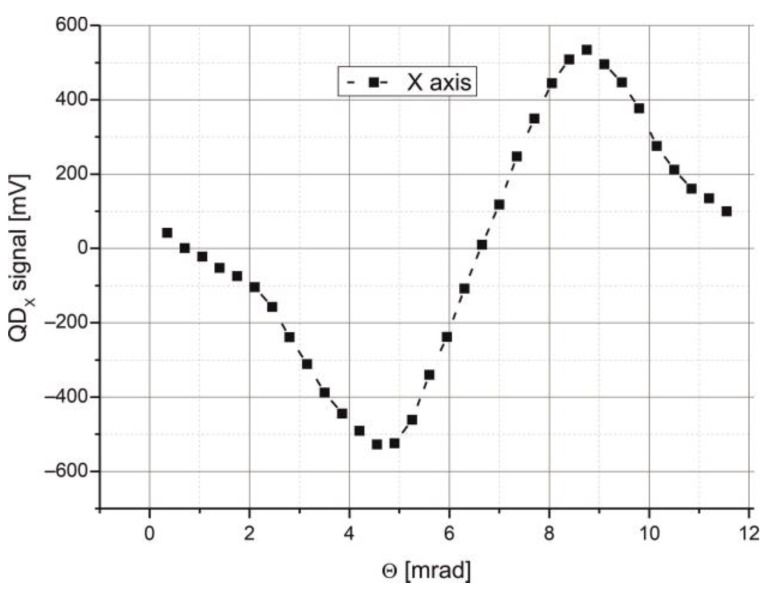
Transfer characteristic of the beam tracking procedure with the *QD*-MWIR module.

The transfer characteristic of the QD-MWIR module during beam tracking was also measured. In this phase of the research, the Picosecond generator again operated in pulse mode. A collimated *QCL* laser beam was focused onto the detector surface using a Ge lens (25 × 40, Edmund Optics). The optical path length was 1 m. Angular adjustments of the beam position were made using a Standa rotary stage (model 8MR-151). Output signals from the *QD-MWIR* module were recorded with a four-channel Tektronix oscilloscope (MSO 6), which also calculated their sum. The axial position difference signal was automatically computed by inputting formula [2] into the oscilloscope math function editor. The resulting transfer characteristic is shown in Figure 14.

Based on this characteristic, the laboratory setup can detect changes in the beam’s angular position with a sensitivity of 320 mV/mrad. However, this sensitivity depends not only on the intrinsic parameters of the *QD-MWIR* module but also on the beam spot size on the detection surface. This issue was previously analyzed and was not the subject of further research. In summary, the results demonstrate that the detection module is capable of monitoring changes in beam position and recording data signals with a signal bandwidth of approximately 110 MHz.

### 4.4. Data Signal Registration

**Figure 15:**
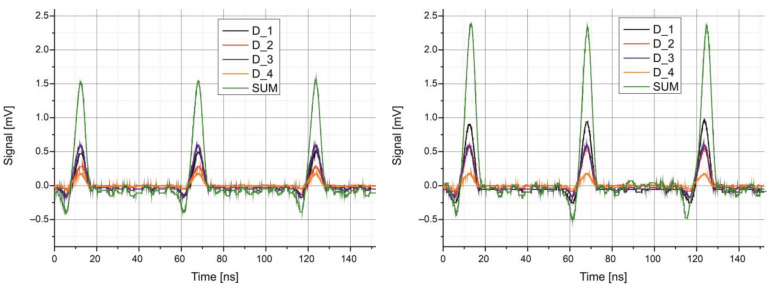
Registered optical signals for two different beam positions on the *QD* detector surface.

The laboratory setup also verified the potential for transmitting pulsed signals using a *QCL* laser-based transmitter. The transmitter generated optical pulses with a duration of 7 ns at a repetition rate of 17 MHz. This configuration enables a minimum data rate of 51 Mb/s, for example, when using PPM modulation. Figure 15 shows the recorded pulse shapes for two different beam spot positions. The shapes are achieved at the optimal operating point of the laser, where both high power and modulation frequency are maintained. These parameters are then used to define the sequence of frame bits and their timing details for further eye diagram tests.

Although the signal amplitudes from individual *QD* detector segments changed with beam displacement, the summed signal remained constant. This characteristic can be effectively used for tracking the direction of the optical beam.

To evaluate the data transmission performance of the FSOC laboratory setup, the Picosecond generator was switched to ‘Pattern mode’. In this mode, it generates a pseudo-random binary sequence (*PRBS*) of pulses with either Return-to-Zero (RZ) or Non-Return-to-Zero (NRZ) encoding. The instrument allows for the random creation of a specified bit frame, with the user defining the number of bits and their positions within the frame. For testing, it was assumed that the 100 ns frame contains 10 bits, each lasting 8 ns. Given the limited duty cycle of the transmitter’s light pulses, only one bit in the frame was assumed to have a value of “1” [0 0 0 1 0 0 0 0 0 0 0 0 0]. A built-in oscilloscope communication analysis tool on the oscilloscope processed the signal recorded by the QD-MWIR module, enabling the identification of the key link parameters.

**Figure 16:**
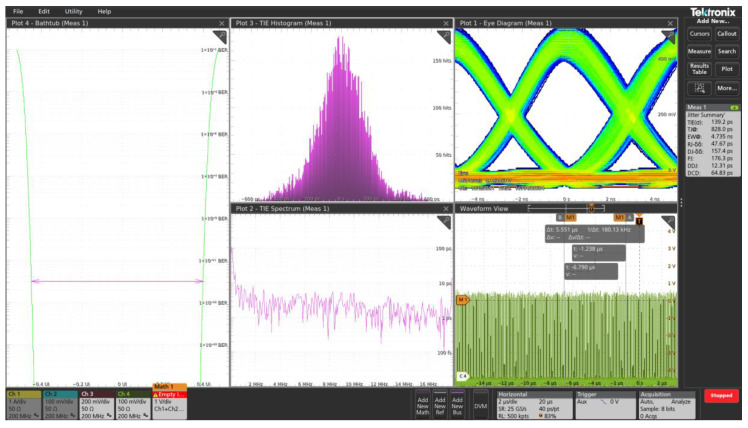
Oscilloscope screenshot of the measurement results.

Figure 16 shows a screenshot of the oscilloscope with the measurement results. The data indicate a minimum time slot for *RZ* (return-to-zero) encoding of approximately 6 ns, and a jitter of less than ±600 ps.

Although these results confirm the system’s ability to record short laser pulses, the main limitation on the available data rate is the low duty cycle (below 5%) of the *QCL* transmitter output.

## 5. Discussion

The paper presents the results of research on a laboratory optical link operating in the *MWIR* range, which could represent a new direction for developing FSO technology. Currently, however, commercial *FSOC* devices utilize radiation in the NIR or SWIR ranges. In real-world conditions, the performance of these links is mainly influenced by their operational environment. For example, when analyzing the effects of scattering—such as when visibility decreases—the use of longer wavelengths reduces radiation attenuation [46,47]. The level of the so-called refractive index structure parameter *C*_n_^2^ defines the influence of atmospheric turbulence. This phenomenon can cause scintillation, beam wandering, and beam spreading. However, the most crucial impact on the link’s parameters is due to scintillations, which describe unpredictable changes in the amplitude and phase of an optical signal propagating in a turbulent atmosphere. These changes are described as the scintillation index:

(12) $$\sigma_{I}^{2}=\frac{\langle I^{2}\rangle -{\langle I\rangle }^{2}}{{\langle I\rangle }^{2}},$$

(13) $$\sigma_{I}^{2}=1.23\times C_{n}^{2}\times k^{\frac{7}{6}}\times R^{\frac{11}{6}}$$

where *k* = 2π/*λ* is the wave number, *λ* is the wavelength, and *R* is the link range. Based on this parameter, the levels of weak (*σ*_I_ < 1), moderate (σ_I_ ∼ 1), and strong (σ_I_ > 1) scintillation are defined. The *σ*_I_ level can generally influence the value of the bit error rate. However, in the *MWIR* range, a smaller deterioration of this parameter occurs compared to *NIR* and SWIR [48,49,50]. When analyzing the influence of background radiation in the *MWIR* range, it turns out that it depends on atmospheric conditions, as with other wavelength ranges. The level of this radiation is slightly lower than that observed in the *SWIR* range, and much lower than in the *NIR* range [51].

The results demonstrate that the developed detection module can be effectively used in *FSOC* systems operating in the *MWIR* range. The use of a *QD* detector enables two functions to be performed simultaneously: data signal acquisition and beam position tracking, both within a bandwidth of 110 MHz.

Integrating these two functionalities significantly simplifies the overall design of the *FSOC* system. Additionally, using the same spectral radiation to perform both functions allows for more accurate compensation of disturbance factors with a spectral origin. Combining signals from the four detector segments also helps reduce the impact of uncorrelated noise, thereby improving the *SNR*. However, analyzing these signals will require more advanced readout electronics equipped with ultra-high-speed *A/D* converters. Future research will focus on developing an integrated platform capable of modulating laser radiation, demodulating the received signal, and detecting beam position changes—specifically for *FSOC* systems operating in the *MWIR* range.

## Figures and Tables

**Table 1:** Comparison of PSD detectors’ performance.

Parameter	L-PSD Detector	QD Detector
Signal	Continuous	Discrete
Resolution	High	Lower
Dynamic range	High	Limited
Speed	High (real-time)	High (real-time)
Signal processing	Advanced	Simple
Acuracy	High	Lower
Costs	High	Lower
Application	High precision tracking, triangulation	Laser pointing, coarse tracking alignment

**Table 2:** Performance comparison of selected *PSD* detectors.

Model	Wavelength Range	Size	Responsivity	Bandwidth (kHz)	Company
PDP90A L-PSD	320–1100 nm	9 × 9 mm	0.6 A/W at 960 nm	15	Thorlabs (Newton, NJ, USA)
PDQ80A QD	400–1050 nm	Ø 7.8 mm	0.64 A/W @ 900 nm	150	Thorlabs (Newton, NJ, USA)
S4349 QD	190–1000	Ø 3 mm	0.45 A/W, 720 nm	20 × 10^3^	Hamamatsu (Hamamatsu City, Japan)
DL2 DL-PSD	400–1100	4 mm^2^	0.4 A/W, 670nm	14 × 10^3^	OSI Optoelectronics (Hawthorne, CA, USA)
J16PS-P6-S10M-HS L-PSD	800–1800	Ø 5 mm	0.6 A/W	Min. 10	Judson (Montgomeryville, PA, USA)
C30927EH-01 Si APD	400–1100	1.55 mm^2^	62 A/W, 900 nm	116 × 10^3^	Excelitas Technologies Corp. (Pittsburgh, PA, USA)
QM-5 QD	3500–6000	4 × 0.2 × 0.2 mm	170 kV/W	Min. 1 × 10^3^	Vigo Photonics Catalogue (Ozarów Maz. Poland)
QM-4 QIP	3000–4500	4 × 0.1 × 0.1 mm	27 kV/W	110 × 10^3^	Vigo Photonics * (Ozarów Maz. Poland)

* described in this paper (Section 3).

**Table 3:** Beam position-sensitive detectors are used in selected *FSOC* systems.

No.	Type of Detector	Wavelength (nm)	Parameter	Speed (kHz)	Refs.
1.	QD InGaAs PIN G6849	1550	Output voltage noise 167 μV	1 × 10^3^	[17]
2.	QD InGaAs Q 3000	1550	Max. position < 0.001 mm	14.5 × 10^3^	[18]
3.	L-PSD DL100-7 PCBA	633	Gimbal σΘ = 34.91 μrad	257	[19]
4.	QD Si	976	x	-	[20]
5.	InGaAs Detector Array	1550	Tracking accuracies σX = 11 μm, σY = 8 μm	0.1	[21]
6.	QD Si QP50-6	520	Tracking speed 2.11 m/s Position error < 4.59 mm (RMS) at a distance of 7.5 m	8.75 × 10^3^	[22,23]
7	IR camera 1920 × 1200	940	Horizontal mobility of 5°/s FOV = 5°	0.163	[24]
8	QD	1310/1550	Pointing error ± 0.05 deg	0.1	[25]
9	CCD	532	Pointing accuracy of 0.5 deg	1.2 × 10^−3^	[26]
10	QD Si	976	Tracking accuracies < 6 μm and 9 μm	4	[20]
11	Si phototransistor cluster	635	x	5.7 × 10^3^	[27]
12	QD	1590/1530	x	5.7 × 10^3^	[28]
13	InGaAs camera (Bobcat 640) PDQ30C (Thorlabs)	1561	Tracking errors < 8 cm (*RMS*) at a range of 1.2 km	0.6	[29]
14	Tracking camera	1550	Tracking accuracy < 20 μrad (RMS) for the airborne terminal Tracking errors < 60 and 40 μrad for the ground station	x	[30]
15	QM-4 QIP (Vigo Photonics)	4050	Transfer coefficient 320 mV/mrad	110 × 10^3^	[*]

* described in this paper (Section 3).

**Table 4:** *MCT* wafer architecture of *PSD-MWIR* detector.

Type	Composition, x	Thickness, d (μm)
CdTe	1	3
N+	0.4	10
N	0.328	0.32
n	0.317	0.8
p	0.312	0.8
P	0.375	0.78
P+	0.464	1.68
n+	0.154	1.9

## Data Availability

The original contributions presented in this study are included in the article. Further inquiries can be directed to the corresponding authors.
